# Site-specific gene knock-in and bacterial phytase gene expression in *Chlamydomonas reinhardtii via* Cas9 RNP-mediated HDR

**DOI:** 10.3389/fpls.2023.1150436

**Published:** 2023-05-19

**Authors:** Hassan Zadabbas Shahabadi, Arash Akbarzadeh, Hamideh Ofoghi, Saeid Kadkhodaei

**Affiliations:** ^1^ Department of Fisheries, Faculty of Marine Science and Technology, University of Hormozgan, Bandar Abbas, Iran; ^2^ Department of Biotechnology, Iranian Research Organization for Science and Technology (IROST), Tehran, Iran; ^3^ Agricultural Biotechnology Research Institute of Iran (ABRII), Isfahan Branch, Agricultural Research, Education and Extension Organization (AREEO), Isfahan, Iran

**Keywords:** *C. reinhardtii*, CRISPR-Cas9, phytase, RNP, NR gene, microalgae, HDR knock-in, genome editing

## Abstract

In the present study, we applied the HDR (homology-directed DNA repair) CRISPR-Cas9-mediated knock-in system to accurately insert an optimized foreign bacterial phytase gene at a specific site of the nitrate reductase *(NR)* gene (exon 2) to achieve homologous recombination with the stability of the transgene and reduce insertion site effects or gene silencing. To this end, we successfully knocked-in the targeted NR gene of *Chlamydomonas reinhardtii* using the bacterial phytase gene cassette through direct delivery of the CRISPR/Cas9 system as the ribonucleoprotein (RNP) complex consisting of Cas9 protein and the specific single guide RNAs (sgRNAs). The *NR* insertion site editing was confirmed by PCR and sequencing of the transgene positive clones. Moreover, 24 clones with correct editing were obtained, where the phytase gene cassette was located in exon 2 of the *NR* gene, and the editing efficiency was determined to be 14.81%. Additionally, site-specific gene expression was analyzed and confirmed using RT-qPCR. Cultivation of the positive knocked-in colonies on the selective media during 10 generations indicated the stability of the correct editing without gene silencing or negative insertion site effects. Our results demonstrated that CRISPR-Cas9-mediated knock-in could be applied for nuclear expression of the heterologous gene of interest, and also confirmed its efficacy as an effective tool for site-specific gene knock-in, avoiding nuclear positional effects and gene silencing in *C. reinhardtii*. These findings could also provide a new perspective on the advantageous application of RNP-CRISPR/Cas9 gene-editing to accelerate the commercial production of complex recombinant proteins in the food-grade organism “*C. reinhardtii*”.

## Introduction

Microalgae encompass a large number of organisms with both prokaryotic and eukaryotic nature, making them an archetypal platform for recombinant technology (Malla et al., 2021). The nutraceutical significance of microalgae is characterized by their rich natural biomolecules and high amount of protein, vitamins, and lipid substances (Potvin and Zhang, 2010; Barrera and Mayfield, 2013; Yan et al., 2016; Bañuelos-Hernández et al., 2017; Ortega-Berlanga et al., 2018; Schmidt et al., 2019; Fayyaz et al., 2020; Sproles et al., 2021). Because of their ability to generate many compounds, microalgae have gained commercial and biotechnological interest, and some species are “generally regarded as safe” (GRAS) for their use as dietary supplements for human and animal food (Rosenberg et al., 2008). Microalgae have many properties that are favorable as a commercial recombinant protein expression system, including fast growth and simple cultivation, with the capability to make post-transcriptional and post-translational modifications (Surzycki et al., 2009). Microalgae also display superior photosynthetic efficiency, being almost three times more efficient in using light than higher plants (Shimizu, 1996). These specifications make these systems an attractive approach for the production of recombinant proteins (Mayfield and Franklin, 2005; Mayfield et al., 2007). *Chlamydomonas reinhardtii* is a green alga, generally recognized as safe by the FDA with many benefits compared to traditional systems for the molecular farming of recombinant proteins (Rosales-Mendoza et al., 2012). These comprise low production price, rapid scalability at the pilot level, lack of human pathogens, and the capability to fold and assemble complex proteins accurately (Rosales-Mendoza et al., 2012). Furthermore, *C. reinhardtii* remains haploid during vegetative growth, and therefore, mutations are almost immediately expressed and specific mutant phenotypes can be easily observed (Shrager et al., 2003).

To date, only three techniques, namely, Zinc Finger Nuclease (ZFN), Transcription-Activator Like Effector Nucleases (TALEN), and Clustered Regularly Interspaced Short Palindromic Repeats (CRISPR), are accessible for efficient genome editing in a specific location (Gupta et al., 2019). In comparison with CRISPR, TALEN and ZFN are more costly and time-consuming (Zhang et al., 2016) and have high off-target mutation tendency and low possibility (Gupta et al., 2014). Eventually, the CRISPR-Cas9 technology offers a simple, easy-to-design, effective, and less expensive method (Li et al., 2013; Walsh and Hochedlinger, 2013; Wang et al., 2013) and can result in a “non-GMO” plant or microalgae (Perez-Pinera et al., 2013). CRISPR, as well as its related protein “CRISPR-associated protein 9” (Cas9), is a method of adaptive immunity in prokaryotes to defend themselves against viruses or bacteriophages (Hille and Charpentier, 2016). Plant genetic engineering has been revolutionized by the prompt development of CRISPR-derived biotechnologies due to their easy, inexpensive, and efficient usage in many plant species (Li et al., 2013; Nekrasov et al., 2013; Shan et al., 2013; Zhang et al., 2019). CRISPR relies on the nuclease activity of CRISPR-associated proteins (Cas) and their particular binding to the genome directed by guide RNAs (gRNAs). CRISPR-Cas creates DNA double-strand breaks and begins endogenous repair pathways (Zhang et al., 2021). CRISPR/Cas9 becomes the most efficient, advantageous, and accurate method of genome editing tool in all living cells and is employed in many applied disciplines (Adli, 2018). Insertions and deletions (Indels) can be presented through the nonhomologous end joining (NHEJ) repair pathway, the dominant repair pathway in plant somatic tissues, leading to random mutagenesis at the target location (Puchta, 2005; Schmidt et al., 2019). Accurate genome editing can be attained through the homology-directed repair (HDR) pathway by introducing homologous repair templates (Zhang et al., 2021). Given the importance of algae in terms of products such as biosequestration of CO_2_, biofuel, bioremediation, cosmetics, aquaculture, agriculture, and recombinant protein production (Mimouni et al., 2012; Markou and Nerantzis, 2013; Skjånes et al., 2013; Cuellar-Bermudez et al., 2015; El Arroussi et al., 2015; Maadane et al., 2015; Gomaa et al., 2016; Minhas et al., 2016), their genomic engineering progress is critical to further gain the microalgae production of high value-added products and bio-energy (Slade and Bauen, 2013). Therefore, wisely used CRISPR/Cas9 tools can help industries to resolve some issues and increase the production yield of some valuable crops. In just a few years of its finding, the CRISPR/Cas9 genome editing technique has already been explored for a large number of uses and had a great impact on the world in many fields including agriculture, medicine, and biotechnology (Bilal et al., 2019).

Nowadays, although the scientific community manages to transform microalgae chloroplast with some successes in order to express desired proteins, nuclear transformation remains really difficult, random, and labor-intensive (Bilal et al., 2019). Homologous recombination can be used in order to insert transgenes during chloroplast transformation, but nuclear transformation remains a more random event (Zhang et al., 2014). Moreover, transgene stability and silencing of the gene of interest (Cerutti et al., 2011; Kim et al., 2015) could also occur, and achievement of such transformation process except in a few cases is difficult or almost impossible (Bilal et al., 2019).

Genomic positional effects, random insertion, and genomic rearrangements are all restrictions that cause researchers to spend a lot of time on screening “the correct transformants” (Bilal et al., 2019). The CRISPR system using the RNA-guided engineered nuclease (RGEN) Cas9 is capable of targeting a specific genomic site thanks to single guide RNA (sgRNA) (Jinek et al., 2012; Cong et al., 2013; Sizova et al., 2013). It appeared as a simpler, versatile, and trustable technique to remove or insert and tune the gene(s) of interest. It is a potent method to edit the nuclear genome knocking-out or knocking-in genes by homology directed DNA repair (HDR) (Mali et al., 2013; Sizova et al., 2013).

Direct delivery of ribonucleoprotein (RNP) complex comprising Cas9 protein and guide RNA has emerged as a strong and prevalent method in the field of CRISPR/Cas genome editing. RNP delivery avoids many of the difficulties associated with mRNA, DNA, or viral delivery, and minimizes off-target effects, insertional mutagenesis, immune responses (Chandrasekaran et al., 2018; Lattanzi et al., 2019), and cellular toxicity (Bloomer et al., 2021). On the other hand, it leads to rapid genome editing due to the elimination of need for intracellular transcription and translation especially for cells with low transcription and translation activity and enhances genome editing efficiencies (DeWitt et al., 2017; Zhang et al., 2021). Moreover, performing *in vitro* cleavage assay with designed gRNAs will save a lot of time, cost, and labor upfront.

In the intestines of monogastric organisms such as humans, birds, aquatic animals, pigs, and other monogastric organisms, there is little or no phytase enzyme production (Turner et al., 2007; Dahiya, 2016). In order to make phosphorus available in the plant diet, phytase produced by microorganisms can be added to the diet (Dahiya and Singh, 2014). The addition of phytase reduces the need to add inorganic phosphorus, and on the other hand, it minimizes the excretion of phosphorus in monogastric organisms (Bali and Satyanarayana, 2001). Moreover, it has been well documented that adding phytase to the diet reduces diet costs by preventing the formation of phytate complexes with minerals (copper, zinc, iron, manganese, etc.), amino acids, fatty acids, starch, and other important components of the diet and subsequently lessens the need for these mentioned factors in the diet (Dahiya, 2016).

Given the advantages of microalgae nuclear expression including protein localization (cytoplasm, nucleus, chloroplast, ER, mitochondria, and secretion) and modifications (phosphorylation, glycosylation, and disulfide bond), and the limitations of expression in the nuclear system of microalgae including genomic positional effects, random insertion, and genomic rearrangements, in the present study, we investigated the RNP-mediated HDR CRISPR system for accurate insertion of the transgene (optimized foreign bacterial phytase gene) at a specific site to achieve stable homologous recombination and consequently reduce the positional effects and gene silencing.

## Materials and methods

### Microalgal strain and culture conditions

The UVM11 “*C. reinhardtii* cell wall deficient strain” was kindly provided by Dr. Ralph Bock and cultured in tris–acetate-phosphate (TAP) medium (Gorman and Levine, 1965; Andersen, 2005) with continuous illumination (250 μmol photons m^−2^ s^−1^), on an orbital shaker (120 rpm) at constant temperature (25°C).

### Preparation of Cas9 protein

*Streptococcus pyogenes* SpCas9 protein expression and purification were briefly performed as follows: SpCas9 expression plasmid pET-28b-Cas9-His (Addgene plasmid, 47327) was transformed into *E. coli* strain Rosetta2 (DE3) and then the expressed protein (Cas9) was purified and concentrated by Nickel NTA affinity chromatography and Amicon (Amicon, 100K MWCO), respectively (Gagnon et al., 2014; Greiner et al., 2017; Yu et al., 2017).

### Target gene and sgRNA design

Target site was selected in the second exon of the *NR* gene (*Nit1*) that encodes nitrate reductase. Designing of sgRNA sites for the target gene (*Nit1*) was done by using the CHOPCHOP v3 web tool (http://chopchop.cbu.uib.no/), which enables us to predict the frameshift rate of each gRNA and evaluate the on-target efficiency along with genome-wide off-targets (Labun et al., 2019). The gene annotation file of *C. reinhardtii* chromosome 9 was achieved from NCBI (Genbank accession number: CM008970.1) and the *Nit1* gene was extracted for submission to CHOPCHOP. Then, all possible sgRNAs on the genomic sequence of *Nit1* were analyzed by CHOPCHOP in CRISPR Cas9 knock-in mode using *Nit1* sequence as the query. Also, in order to confirm the presence of suitably designed sgRNAs in *C. reinhardtii* uvm11 strain, *Nit1* gene was PCR amplified (795 bp PCR product) and partially sequenced (Genbank accession number: OP566529) by primers T12-F and T12R (1). One selected gRNA was 20 base pairs (bp) long, followed by a protospacer adjacent motif (PAM)–NGG that is located in the second exon; their detailed information is presented in **Supplementary Table 1**.

### *In vitro* guide RNA synthesis

The sgRNA DNA template, which had a T7 (5’-TAATACGACTCACTATA-3’) promoter sequence, followed by the 20-base target-specific gRNA sequence without the PAM and a tracrRNA sequence, was assembled by overlapping primers (Gagnon et al., 2014; Yu et al., 2017; Hu et al., 2019; Kang et al., 2020). Two oligonucleotide primers F-gr2 and R-gr123 (**Supplementary Table 2**) having 18 nt overlap were synthesized (GenScript) and then assembled with the following thermal cycling program: 98°C for 1 min, 98°C for 20 s (denaturation), 54°C for 20 s (renaturation), 68°C for 50 s (extension), and 68°C for 5 min (final extension). PCR cycles were repeated 25 times (**Supplementary Table 3**). The PCR products (gRNA DNA template) were gel purified and 75 ng of the template DNA was used for *in vitro* gRNA transcription, using the Hi Scribe T7 Quick High Yield RNA Synthesis Kit (New England Biolabs) at 37°C overnight following the manufacturer’s protocol (NEB #E2050).

### Donor plasmid construction for CRISPR-based gene knock-in

**Figure 1:**
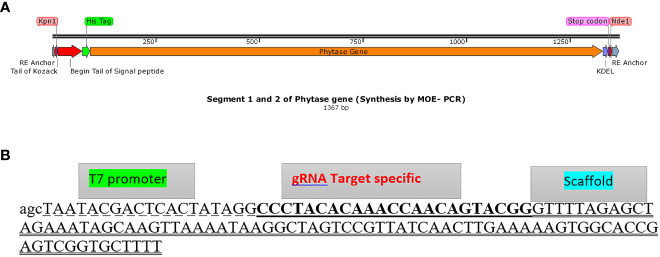
Assembly of phytase gene and gRNA DNA construct template. **(A)** The phytase gene construct comprising parts 1 and 2 (anchor, Kpn1 restriction enzyme, Kozack, signal peptide, His tag, codon-optimized phytase gene, KDEL, Nde1 restriction enzyme, and anchor, respectively, from the upper part to the lower part) assembled through MOE-PCR for expression in *C*. *reinhardtii* (Genbank accession number: OP566531). **(B)** gRNA DNA template (gDNA) assembled sequence (125 bp) including T7 promoter (dashed format), gRNA target plus PAM (underlined format), and scaffold (two underlined format).

First, the gene of interest (bacterial phytase gene) sequence in terms of critical parameters affecting the efficiency of gene expression, including codon usage, GC content, CpG dinucleotides content, mRNA secondary structure, cryptic splicing sites, premature polyA sites, internal chi sites, ribosomal binding sites, negative CpG islands, RNA instability motif (ARE), repeat sequences (direct repeat, reverse repeat, and dyad repeat), and restriction sites that may interfere with cloning, was optimized for translation in *C. reinhardtii* and then synthesized by GeneScript (Genbank accession number OP566532). Using the designed specific overlapping primers “F-SE1, R1-SE1, and R2-SE1”, the fragment Kpn1 restriction site, microalgae-specific signal peptide, His-tag at the 5’ end (part 1 of the phytase gene), and specific overlap primer R-SE2 (**Supplementary Table 2**) for *Nde1* restriction site and KDEL at the 3’ end of the phytase gene sequence (part 2 of phytase gene) were assembled through MOE-PCR (Kadkhodaei et al., 2016) in two independent PCR reactions, respectively (**Supplementary Tables 4**, **5**; **Figure 1A**). The construct prepared by PCR (Genbank accession number: OP566531) was then cloned to the pChlamy3 expression vector (**Supplementary Figure 1**) using *Kpn1* and *Nde1* restriction enzymes.

In the next step, specific primers F-INC2 and R-INC2 (**Supplementary Table 2**) were designed in a way to have overlapping ends with homologous arms (left arm: HSP70A inducible promoter; right arm: RBCS2 3’UTR regions) of the phytase gene construct. The construct was amplified through PCR, and the PCR product was named as the inner construct (Genbank accession number: OP566530). The left and right homologous arms (Genbank accession numbers: OP595611 and OP595612) required for CRISPR/Cas9-mediated HDR were selected according to both sides of the Cas9 cleavage site in a length of approximately 1,000 nt. The homologous arms were PCR amplified using the relevant overlapping primers F-HU2, F-HD2 and R-HU2, R-HD2 (**Supplementary Table 2**) with the inner construct, as well as adding the gRNA and PAM sequences at the beginning of both F-HU2 forward and R-HD2 reverse primers.

The p1ChlamyCK-I/O vector backbone including origin of replication (ori) and the genes encoding ampicillin and hygromycin resistance along with the regulatory elements were amplified from pChlamy3 vector backbone (**Supplementary Figure 1**) using primers F-pCh2 and R-pCh2 (**Supplementary Table 2**) containing overlapping sequences with the left and right homologous arms accordingly.

Finally, to generate the donor plasmid for the CRISPR knock-in system (p1ChlamyCK-I/O), all amplified fragments, including the inner construct, left and right homologous arms, and pChlamy3 backbone, were gel purified, assembled through MOE-PCR (**Supplementary Table 6**), and subsequently transformed to *E. coli* (DH5α) for future experiments (**Supplementary Figure 2**). In order to confirm the accuracy and integrity of the assembled p1ChlamyCK-I/O donor plasmid, both the restriction mapping (using one or two restriction sites, *HindIII*, *BamHI*, *XhoI*, *SapI*, and *EcoRv*, embedded at the flanking regions of the fragments) and sequencing (through primer walking) (**Supplementary Table 7**) were applied.

### Preparation of RNP complex and *in vitro* cleavage assay

The activities of the sgRNAs and the Cas9 protein were verified *in vitro* prior to *in vivo* experiments. The p1ChlamyCK-I/O donor plasmid assembled in the previous steps was targeted as DNA template. *In vitro* cleavage assay was performed according to Hu et al. (2021) with some minor modifications (**Supplementary Table 8**): in the optimized condition to obtain the RNP complex, 1,300 ng of sgRNA and 130 ng of Cas9 protein (10:1 ratio) were pre-mixed at 37°C for 15 min along with 3 µl of 10X Cas9 activity buffer and nuclease-free water to a final volume of 15 µl. Then, for each experiment, 400 ng of the p1ChlamyCK-I/O donor plasmid was added in the above reaction, and the mixtures were incubated at 37°C for 4 h. To inactivate the Cas9 nuclease, the reactions were incubated at 65°C for 10 min. The final products to confirm the activity and correctness of enzyme cleavage were separated by 0.8% agarose gel electrophoresis.

### Transformation of *C. reinhardtii*

Direct delivery of the RNP complex and the donor plasmid to *C. reinhardtii* (strain UVM11) was carried out using the glass bead method as described by Picariello et al. (2020) and Neupert et al. (2012) with minor modifications. Glass beads, 425–600 µm in diameter (Sigma-Aldrich), were washed using concentrated sulfuric acid, then rinsed thoroughly with distilled water for several times, dried, and sterilized by autoclaving. The glass beads (300 mg), the donor plasmid linearized by *Sca*1 enzyme (1µg), the RNP complex (1.3 µg of Cas9 plus 13 µg of gRNA under *in vivo* conditions), and 300 µl of *C. reinhardtii* cells (harvested at a density of 4×10^6^ cells/ml and concentrated to 3×10^8^ cells/ml) were added to the tube for knock-in experiments. The tube was vortexed at a maximum speed for 20 s, rested for 10 s, then vortexed again at the top speed for 15 s. Also, two control samples were considered in glass bead transformation experiments, one containing the donor plasmid without the RNP complex, and the other lacking both the donor plasmid and the RNP complex.

### Post-transformation cell recovery, plating, and selection of the transgenic cells

Cell recovery and plating were carried out following the method of Picariello et al. (2020) with minor optimization. Transformed cells were placed on an orbital shaker (70 rpm) under dim lights for 24 h at 25°C. Finally, 75 µl (for spread culture) or 25 µl (for spot culture) of the transformed cell culture was harvested and plated on a solid TAP medium supplemented with 10 µg/ml hygromycin. The plates were incubated at 25°C under continuous light (250 μmol photons m^−2^ s^−1^) until visible colonies appeared (approximately between 10 and 14 days). Finally, positive cell lines (grown on 10 µg/ml hygromycin) were picked for colony maintenance and genome editing verifications including PCR, sequencing, and qPCR.

### Algal mutant screening procedures

#### Verification of knocked-in mutant by PCR amplification and sequencing analysis

Genomic DNA was extracted from positive colonies (0.5 mm in size) using 500 µl of 2x CTAB (0.1 M Tris-HCl, 0.02 M EDTA, 1.4 M NaCl, 2% CTAB, pH 8.0, and 2% PVP). The target regions including 1,332 bp (specific primer: F-SE1 and R-SE1) of the inserted gene (phytase), a 3,047-bp (specific primer: T12-F and T12-R) fragment comprising gRNA sequence (593 bp upstream of the gRNA sequence, 2,252 bp inserted phytase gene along with the regulatory elements, and 202 bp downstream of the gRNA sequence) in the genome, and 3,753 bp (specific primer: 3327-F and T12-R) of the donor plasmid (1,299 bp upstream of the gRNA sequence along with the regulatory elements of plasmid, 2,252 bp inserted phytase gene along with the regulatory elements, and 202 bp downstream of the gRNA sequence) were amplified by PCR using the specific primers (**Supplementary Table 2**).

All in all, the 1,332-bp fragment was used to confirm the presence of the phytase gene in the positive colonies. The 3,047-bp and 3,753-bp fragments were used to validate the editing that occurred in exon 2 of the nitrate reductase gene (correct editing/knock-in) and the absence of donor plasmid residues (incorrect editing or false-positive results due to the presence of plasmid residues), respectively. The 3,047-bp PCR product was gel purified and sequenced using Sanger sequencing by Gene Fanavaran Co. (Tehran, Iran). The multiple sequencing contigs were then aligned and assembled using Geneious software (2020.2.5) to verify the integrity of the inserted constructs in the correct position (accurate knock-in editing) as well as the possible mutations.

#### Phytase expression analysis by RT-PCR and qRT-PCR

Total RNA was extracted from WT and *NR* knocked-in mutants using the TRIzol (YTA Co., Iran Cat No: YT9066) reagent and cDNA was synthesized according to the manufacturer’s (YTA Co., Iran Cat No: YT4500) instructions.

The RT-PCR assay was performed by the Bio-Rad PCR cycler system as follows: 4 min at 98°C; 30 cycles of 30 s at 98°C, 20 s at 60°C, 30 s at 68°C, and 5 min at 68°C for final extension.

The qPCR assays were performed using YTA SYBR Green qPCR MasterMix 2X (YTA Co. Iran Cat No: YT2551) by a Mic qPCR Cycler system. The thermal profile used for qPCR was as follows: 3 min at 95°C; 25 cycles of 10 s at 95°C, 10 s at 60°C, and 20 s at 72°C. Each reaction contained 1 µl of the undiluted cDNA and a reaction master mix containing 2x SYBR Green qPCR Mix with ROX dye and 0.2 µM of each primer. All qPCR assays were run with appropriate controls including the Non-Template Control (NTC).

For RT-PCR and qRT-PCR, one set of GOI primers (Fq-Phytase and Rq-Phytase) (**Supplementary Table 2**) was used to amplify and quantify a region of approximately 221 bp in the inserted phytase gene. For the control, CBLP gene was used as the housekeeping gene (Moseley et al., 2006; Allen et al., 2007; Zhao et al., 2009), and the primers F-CBLP and R-CBLP (**Supplementary Table 2**) were used to amplify a region of approximately 221 bp in size.

### Growth analysis

In order to evaluate whether the knock-in strategy has no negative effect on the cell growth, both WT and knocked-in *C. reinhardtii* cells were grown in 100 ml of TAP media illuminated with continuous light (250 μmol photons m^−2^ s^−1^) on an orbital shaker (120 rpm) at 25 °C. The initial cell concentration for each culture was 1× 10^5^ cells/ml. The comparative analysis of growth parameters was performed according to cell count (sampling of 1 ml culture each day) by using a hemocytometer and light microscopy for a period of 7 days.

## Results

### RNP complex preparation and *in vitro* cleavage assay optimization

The presence of expressed and purified Cas9 nuclease was evaluated by SDS-PAGE in the eluted and concentrated fractions, which showed a high degree of purity and correct size. Based on measurement using the Bradford method and the open-source image processing program “Image J” (National Institutes of Health, Bethesda, MD), approximately 215 µg/ml Cas9 endonuclease was yielded from the bacterial culture medium containing the Cas9-expressing plasmid (pET-28b-Cas9-His) (https://www.addgene.org/47327/sequences/).

The DNA template of gRNA was assembled using two synthesized oligonucleotides (**Supplementary Table 2**) by overlapping PCR (**Figure 1B**) and the correct assembly was confirmed on 1.5% agarose gel. The results showed successful fusion of the two fragments with 120 bp amplicon size. Subsequently, *in vitro* gRNA synthesis using the relevant DNA template resulted in the production of 100 mg/ml gRNA, and overall qualitative (1.5% agarose gel and nanodrop data) and quantitative (nanodrop data) results verified the correct gRNA synthesis.

Donor DNA fragment containing two flanking gRNA target sequences (**Supplementary Figure 2**) was used to evaluate the efficiency and activity of the Cas9/gRNA complex through *in vitro* cleavage assay. In order to optimize RNP complex preparation and cleavage assay, various amounts of Cas9 and gRNA were tested. The optimal concentration of gRNA and Cas9 for complete cleavage of the target was determined as 1,300 ng and 130 ng, respectively. The *in vitro* cleavage assay clearly showed the expected size of the amplicons (4,000 and 3,500 bp), while there was no cleavage seen when only gRNA was added to the target template.

### Donor plasmid assembly for the CRISPR-based knock-in

We designed a modular donor plasmid “p1ChlamyCK-I/O” (Genbank accession number: OP236418) (**Supplementary Figure 2**) for CRISPR-based knock-in that uses the HDR pathway to target *NR* Gene (Exon2) in *C. reinhardtii*. Briefly in this system, 12 primer pairs (**Supplementary Table 2**) were designed through *in silico* screening by Oligo Analyzer software. The MOE PCR assembly method was used to join all fragments, respectively, including vector backbone (hygromycin, ampicillin, and ori sequence), upstream gRNA target sequence and the PAM site, left homologous arm, GOI cassette (inducer-promoter-intron-signal peptide-His tag-phytase gene-KDEL-terminator), right homologous arm, and downstream gRNA target sequence containing the relevant PAM site (**Supplementary Figure 2**).

The restriction mapping (by using HindIII, EcoRv, Sap1, Xho1, and BamH1 enzymes), *in vitro* cleavage assay (by using RNP complex), and sequencing by primer walking (Genbank accession number: OP236418) confirmed the precise assembly of the donor plasmid (p1ChlamyCK-I/O), which ensures accuracy of the knock-in process in *C. reinhardtii*.

### Direct delivery of knock-in RNP complex to microalgal cell (*C. reinhardtii* UVM11 strain)

**Figure 2:**
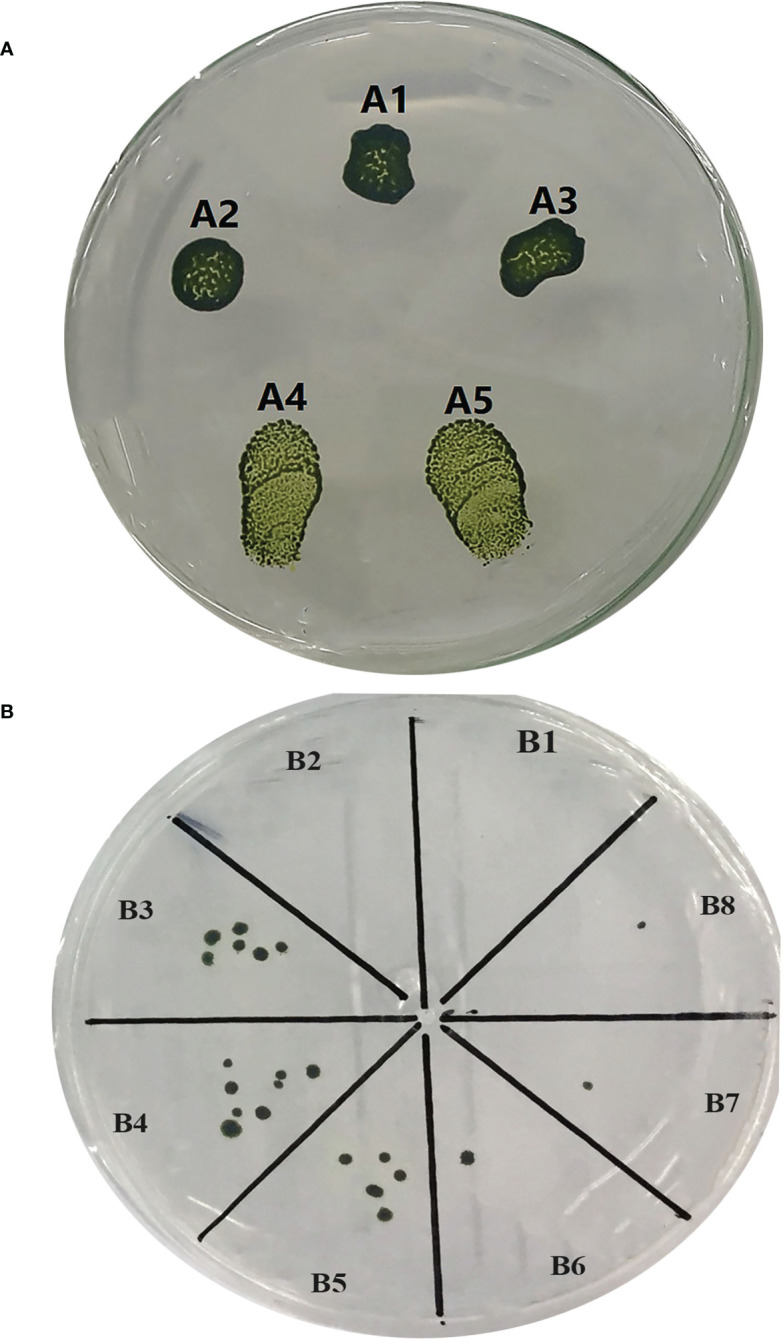
Culture of the transformed and untransformed *C*. *reinhardtii*. **(A)** Spot culture of the transformed and untransformed *C*. *reinhardtii* on selective TAP medium. (A1) Spot culture of the treated *C*. *reinhardtii* under glass bead transformation conditions but without using the donor plasmid and the RNP complex. (A2) Spot culture of the treated *C*. *reinhardtii* under glass bead transformation conditions only using the donor plasmid “p1ChlamyCK-I/O”. (A3) A2 repeat. (A4) Spot culture of the treated *C*. *reinhardtii* under glass bead transformation conditions using the donor plasmid “p1ChlamyCK-I/O” and the RNP complex. (A5) A4 repeat. **(B)** The *C*. *reinhardtii*-positive colonies obtained using the glass bead transformation method on the selective TAP medium supplemented with 10 µg/ml Hygromycin B. (B1) *C*. *reinhardtii* without any transformation. (B2) Treated *C*. *reinhardtii* under transformation conditions but without using the donor plasmid and the RNP complex. (B3) Treated *C*. *reinhardtii* under transformation conditions using the donor plasmid “p1ChlamyCK-I/O” and the RNP complex. (B4, B5) B3 repeat. (B6) Treated *C*. *reinhardtii* under transformation conditions using only the donor plasmid “p1ChlamyCK-I/O”. (B7 and B8) B6 repeat.

In this research, three independent transformations with two repeats (**Supplementary Table 9** and **Figures 2A, B**) were performed and putative positive colonies were screened on the TAP agar medium supplemented with 10 µg/ml hygromycin B. Overall, 162 positive transformants were selected by the glass beads transformation method with the transformation efficiency of 2,070 cfu/μg of the knocked-in *C. reinhardtii*-positive colonies using the donor plasmid “p1ChlamyCK-I/O” and the RNP complex compared to the control (without RNP complex and only using the donor plasmid “p1ChlamyCK-I/O”) with 1,511 cfu/μg. The concentrations of the cell working solution was standardized to 330 million cells ml^−1^ and 300 µl of this working solution was used in a single transformation resulting in 2,070 cells per transformation reaction. The concentrations were based on the comparison of the cfu between the transformation reactions carried out using 100 × 10^6^ cells together with 1 µg of the donor DNA and determined concentrations of RNP complexes (1.3 µg:13 µg or 10-fold of optimized *in vitro* Cas9:gRNA ratio) for the knock-in.

**Figure 3:**
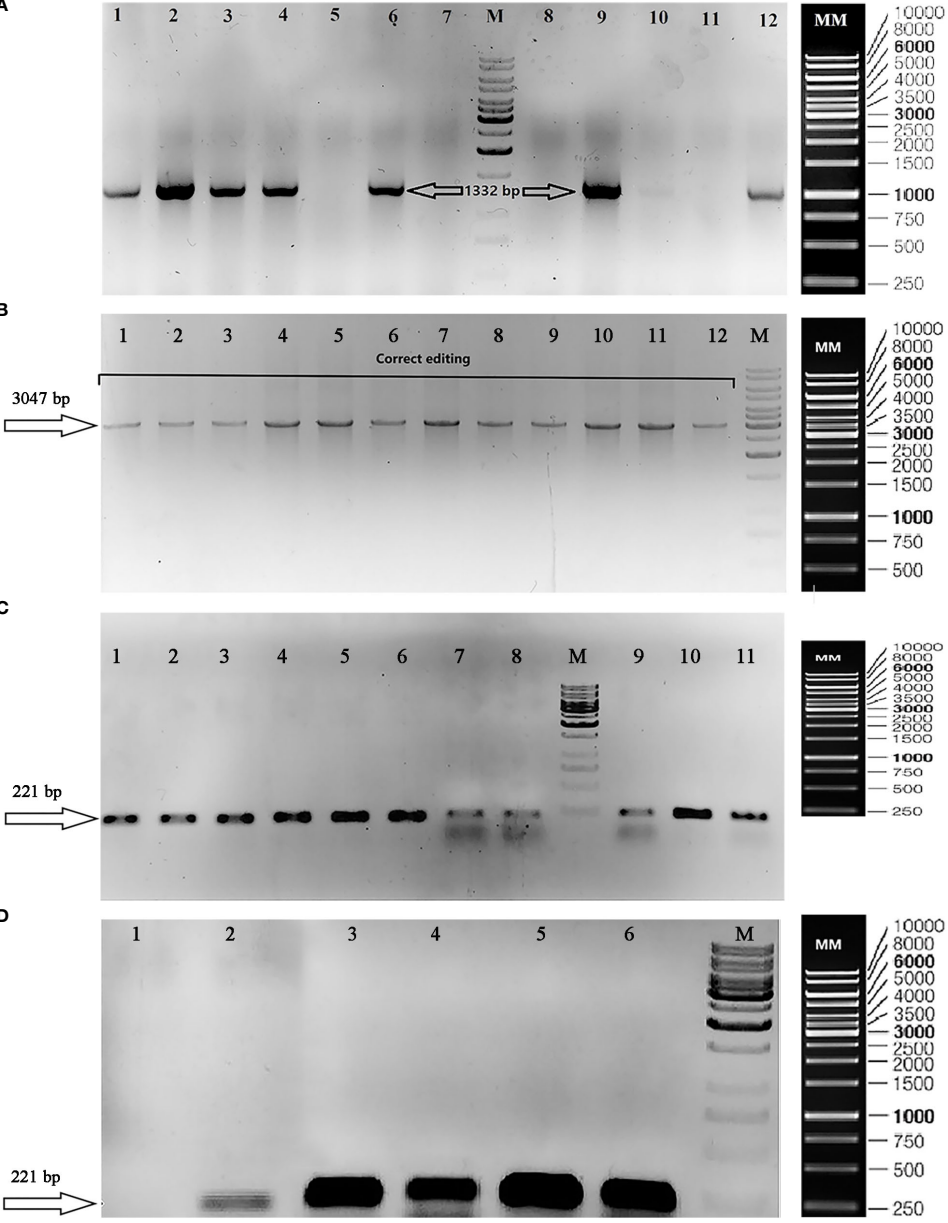
Gel electrophoresis to validate transformation, correct editing, and transcription of phytase gene. **(A)** TAE agarose gel (1%) showing confirmation of the presence of phytase gene (1,332 bp/Genbank accession number: OP566531) in the positive colonies using FSE1 and RSE2 primers. The lane numbers 1, 2, 3, 4, 6, 9, 10, and 12 are representative of the positive colonies (existence of phytase gene: 1,332 bp); (M) DNA size marker [GeneRuler 1 kb DNA Ladder, ready-to-use (Catalog Number SM0313)]; (MM) Marker Map of GeneRuler 1 kb DNA Ladder, ready-to-use (Catalog Number SM0313). **(B)** TAE Agarose gel (1%) showing knocked-in phytase gene at the desired position (NR gene–Exon2) by PCR using FT12 and RT12 primers. (M) DNA size marker [GeneRuler 1 kb DNA Ladder, ready-to-use (Catalog Number SM0313)]; (MM) Marker Map of GeneRuler 1 kb DNA Ladder, ready-to-use (Catalog Number SM0313). **(C)** TBE Agarose gel (1%) showing RT-PCR products including the following: Colonies 1–11 containing the correct editing using Fq-Phytase and Rq-Phytase primer (insertion of the phytase gene cassette at the NR target site); (M) DNA size marker [GeneRuler 1 kb DNA Ladder, ready-to-use (Catalog Number SM0313)]; (MM) Marker Map of GeneRuler 1 kb DNA Ladder, ready-to-use (Catalog Number SM0313). **(D)** TBE Agarose gel (1%) showing RT-PCR products including the following: (1) wild-type colony using Fq-Phytase and Rq-Phytase primers to confirm phytase gene expression; (2) wild-type colony using F-CBLP and R-CBLP primers to confirm reference gene expression; (3) colony 215 [correct edited sample (transformation using RNP complex and the donor plasmid “p1ChlamyCK-I/O”)] using Fq-Phytase and Rq-Phytase primers to confirm phytase gene expression; (4) colony 153 [correct edited sample (transformation using the RNP complex and the donor plasmid)] using Fq-Phytase and Rq-Phytase primers to confirm phytase gene expression; (5) colony 215 using F-CBLP and R-CBLP primers to confirm reference gene expression; (6) colony 153 using F-CBLP and R-CBLP primers to confirm reference gene expression; (M) DNA size marker [GeneRuler 1 kb DNA Ladder, ready-to-use (Catalog Number SM0313)]; (MM) Marker Map of GeneRuler 1 kb DNA Ladder, ready-to-use (Catalog Number SM0313).

Three specific primer pairs were used to verify the positive colonies (**Supplementary Table 2**) including FSE1 and RSE2 to confirm the presence of the phytase gene (Genbank accession number: OP566531) (**Figure 3A**), FT12 and RT12 to validate the correct edit in exon 2 of the nitrate reductase gene (Genbank accession number: OP236417) (**Figure 3B**), and F3327 and RT12 to confirm the absence of primary p1ChlamyCK-I/O residues with a false-positive result.

PCR-based verifications revealed that more than 65% of colonies (106 out of 162 selected colonies showed resistance to hygromycin) contained the complete phytase gene cassette (Signal peptide, His6 tag, Goi, KDEL) (1,332 bp) (Genbank accession number: OP566531) and 52 colonies contained a 3,047-bp fragment gRNA target sequence among which 28 and 24 colonies showed incorrect (presence of the p1ChlamyCK-I/O residues) and correct (insertion of the phytase gene cassette at the *NR* target site) editing, respectively (**Supplementary Table 9**). In the present study, 24 colonies containing the correct editing included sample numbers 4, 10, 56, 58, 65, 78, 80, 81, 82, 83, 116, 117, 131, 138, 148, 150, 152, 153, 191, 215, 224, 238, 265, and 277, and colony number 332 was considered as control sample (transformation without using the RNP complex and only using the donor plasmid “p1ChlamyCK-I/O”). In general, the editing efficiency of ~15% was observed in the present study. The PCR products obtained from the representative correct edited colonies were further analyzed and confirmed by sequencing (Genbank accession number: OP236417). Sequencing of the target fragment clearly demonstrated integrity and correct insertion of the gene cassette (2,252-bp fragment containing Hsp70-Rbcs2 promoter, Rbcs2 intron1, Signal peptide, His6 tag, Goi, KDEL, and 3UTR Rbcs2) into the exon 2 of the *NR* gene in *C. reinhardtii*.

### RT-PCR and qRT-PCR analysis of the foreign gene expression

Phytase transcription was confirmed by RT-PCR (**Figures 3C, D**) and Ct values of qRT-PCR to verify the inserted foreign gene expression at the RNA level (**Supplementary Figure 3**, **Supplementary Table 10**). As shown in **Figures 3C, D**; **Supplementary Figure 2**, and **Supplementary Table 10**, the inserted foreign gene was successfully transcribed. The resulting candidates were then verified by Sanger sequencing and phenotypic analysis. Finally, results of the present study showed that the phytase gene was successfully transcribed at the desired target site with constant expression level in correct editing samples (sample numbers 215, 153, and 81) and transient expression for two generations in control sample (sample number 332).

### Growth analysis

To examine whether the knock-in process negatively affects *C. reinhardtii* cells’ growth, the genome-edited samples were analyzed for growth curves. The growth of knocked-in *C. reinhardtii* was similar to that of the wild type and the cell growth of both WT and knocked-in samples was limited to approximately 15 × 10^6^ cells ml^−1^.

## Discussion

Microalgae have lately been getting attention as a potential low-cost bio-factory for the production and development of a wide range of commercial products including nutraceuticals, animal feeds, therapeutics, industrial biochemicals, and biofuels (Olaizola, 2003; Pulz and Gross, 2004; Spolaore et al., 2006; Chisti, 2008; Christaki et al., 2011; Jones and Mayfield, 2012). Genetic engineering could facilitate and ensure sustainable and higher yields of the targeted algae-based value-added products (Walker et al., 2005; Raja et al., 2008; Radakovits et al., 2010; Specht et al., 2010; Gong et al., 2011; Georgianna and Mayfield, 2012; Gimpel et al., 2013). Therefore, microalgae are able to revolutionize various industries such as nutrition (food and feed), health, energy, and biochemicals in particular. However, the main barriers in the production of beneficial compounds from algae strains are either the necessity of efficient molecular tools or the low expression level of heterologous genes. Transgene silencing, genomic positional effects, random insertion, transient expression, and genomic rearrangements are among the major challenges and limitations in genome engineering of *C. reinhardtii* (Cerutti et al., 1997; Fuhrmann et al., 1999; Rasala et al., 2012). These obstacles can be resolved by the recently developed gene-editing techniques. The new molecular genetic tools such as the CRISPR/Cas systems could be efficiently used for the remarkable development of the microalgae current state in terms of genetic, metabolic, and pathway engineering (Behler et al., 2018; Jagadevan et al., 2018; Naduthodi et al., 2019; Patel et al., 2019; Sreenikethanam et al., 2022; Jeong et al., 2023) and finally impact the improvement of transgenic algae as a cell bio-factory.

Here, for the first time in microalgae, we reported insertion of the codon-optimized prokaryotic phytase gene from bacterium *Buttiauxella* sp. by the Cas9 RNP-mediated knock-in through the homology-directed repair (HDR) in the *Nit1* gene of the eukaryotic microalgae *C. reinhardtii*. We targeted exon 2 from the *Nit1* gene that encodes the nitrate reductase, which catalyzes the reduction of nitrate to nitrite (Plecenikova et al., 2013), to knock-in microalgae optimized bacterial phytase gene in this insertion site in *C. reinhardtii* using the HDR CRISPR-Cas9 system. As a result, after knock-in GOI in this insertion site, microalgae are not able to consume nitrate, but they have the ability to absorb and consume nitrite and ammonium without any problems to continue the growth and life (Fernandez and Galvan, 2008; Sanz-Luque et al., 2015; Wang et al., 2016; Bellido-Pedraza et al., 2020; Krishnan et al., 2020; Kumar and Bera, 2020; Salbitani and Carfagna, 2021).

In the present study, *in vivo* assays were optimized to utilize very smaller amounts of gRNA and Cas9 (1.3 μg:13 μg) in comparison with the quantities already reported for gene knock-out in *C. reinhardtii* (Baek et al., 2016). Baek et al. (2016) utilized 200 μg of Cas9 protein and 140 μg of *in vitro* transcribed gRNA for direct delivery into *C. reinhardtii*, which seems to be very high concentrations and the production of such quantities is difficult and costly.

In this study, several gRNAs were designed (**Supplementary Table 1**) to target *Nit1* coding sequence among which the ones targeting preferably 5’-end exons showed the highest efficiency while the lowest off-targets were screened *in silico*. The designed gRNA targeting exon 2 was assessed *in vitro* to verify its cleavage efficiency of the target sequence. For *in vitro* cleavage assay, we used various concentration ratios of Cas9:gRNA (**Supplementary Table 8**) in which 130:1300 ng was indicated as the optimized ratio, which succeeded in cutting both targeting sites embedded in the synthesized p1ChlamyCK-I/O donor plasmid (400 ng) with high accuracy and efficiency. In other studies, the range of Cas9 and gRNA used for *in vitro* cleavage assays has been reported as 150–600 ng and 100–500 ng for Cas9 and gRNA, respectively, using approximately 100 ng of target DNA (Shin et al., 2016; Dhokane et al., 2020; Kang et al., 2020). In the present study, a lower amount of Cas9 protein was used to cut a larger amount of target DNA with two cutting sites, while the amount of gRNA was higher. Considering four times more target DNA amount used and also the presence of double cutting sites, it indicates that a smaller amount of gRNA has been used. The results of the *in vitro* assay demonstrated the efficient quality of the synthesized gRNA, purified Cas9, and the assembled RNP complex on one hand and the high efficiency of the designed gRNA in identifying the target DNA for CRISPR knock-in operations, on the other hand.

The bacterial phytase gene sequence was first optimized and synthesized (Genbank accession number: OP566532) (GenScript). In the second step, the p1ChlamyCK-I/O donor plasmid (Genbank accession number: OP236418) segments were successfully assembled (**Supplementary Figure 2**) by MOE-PCR (Kadkhodaei et al., 2016), and then different amounts of Cas9 and gRNA along with a fixed amount of the p1ChlamyCK-I/O donor plasmid were transferred to the algal cell. In three independent transformations carried out with two replicates, we obtained 162 positive transformants on TAP agar medium containing 10 µg/ml hygromycin B. Considering the amount of plasmid used (1 µg), the cell density loaded on the selective TAP medium (13 × 10^6^ cells/ml), and the ratio of *in vivo* Cas9:gRNA (1.3 µg:13 µg or 10-fold of optimized *in vitro* Cas9:gRNA ratio), the transformation efficiency of 2,070 cells was obtained per reaction (**Supplementary Table 9**). In the study conducted by Kindle (1989) using the same glass bead method (Kindle, 1990), the efficiency of the transfer was 1,000 cells, but in the present study, it is more than twofold. In another study conducted by Yamano et al. (2013) using the electroporation method on different *C. reinhardtii* strains, the transformation efficiency was reported to be between 150 and 7,614 cells; our results fall within this range. In general, and according to the obtained results, the glass bead method is a widely used alternative for the delivery of macromolecules into *C. reinhardtii* cells (Kindle, 1990) and is highly economical compared to the electroporation method, which is not readily available.

In order to score the efficiency of the gene knock-in through Cas9 RNP-mediated HDR and targeting *Nit1*, PCR analysis of all selected positive colonies (162 colonies) were isolated from selective medium-screened 106 (65.5%) samples containing phytase gene. Finally, after further molecular investigations through PCR and sequencing, 24 clones with correct editing were obtained, wherein the phytase gene cassette was located in exon 2 of the *Nit1* gene, showing an editing efficiency of approximately 14.81%. The efficiency of correct editing in the present study was higher than the previous studies published by Baek et al. (2016); Shin et al. (2016), and Ferenczi et al. (2017) with efficiencies of 0.5%, 1.4%, and 10%, respectively (Baek et al., 2016; Shin et al., 2016; Ferenczi et al., 2017), and almost equal to Greiner et al. (2017) findings (14.8%) (Greiner et al., 2017). This indicated the reasonable values obtained in our study compared to previous studies conducted through CRISPR/Cas9-mediated HDR. In a study conducted by Kim et al. (2020), the editing efficiencies were reported to be between 16.5% and 36.8% (Kim et al., 2020), which could be attributed to the location of the insert, the nature of the transgene sequence, the size and sequence of the homologous arms, etc. Also, in the study conducted by Movahedi et al. (2022), using gene silencing of XRCC4 [inhibitor of non-homologous end joining (NHEJ) recombination cofactor] with a combination of CtIP (HDR enhancer factor) and MRE11 (HDR enhancer factor) overexpression, an efficiency of 48% was obtained (Movahedi et al., 2022). In general and according to the objective of the present study, obtaining smaller number of clones with correct editing and with stable and continuous expression of the target recombinant protein (phytase) would be reasonable.

Furthermore, in our work on phytase gene knock-in and genome editing in *C. reinhardtii*, cultivation and selection operations on correct positive edited colonies (24 colonies) were carried out during 10 generations in a period of 100 days, and the results showed correct and stable editing, while the samples transformed by the traditional method (without using RNP complex or CRISPR method) lost the phytase gene after two generations.

To confirm the efficiency of the knock-in of interest, RT and qRT-PCR analysis showed the presence of the knocked-in external phytase gene and proved the gene transcription in the edited colons (**Figures 3C, D** and **Supplementary Figure 3**). Also, the Ct values (**Supplementary Table 10**) obtained from the qPCR results for the internal reference gene and the external phytase gene were almost the same. Since the reference genes usually have continuous and high expression level, it could be concluded that the phytase gene has relatively reasonable expression at the RNA level. On the other hand, continuity of the expression even in the 10th generation of the knocked-in clones could prove the phytase expression stability in the *NR* (nitrate reductase) site and also the suitability of the *NR* site for the insertion of foreign genes of interest without being affected by gene silencing.

## Conclusion

To our knowledge, this is the first successful report of CRISPR/Cas9 RNP-mediated knock-in of the bacterial phytase gene in the *NR* gene of *C. reinhardtii* with approximately 15% editing efficiency. Furthermore, cultivation and selection were carried out on all 24 positively edited colonies in 10 generations during a 100-day period and results indicated correct and stable knock-in editing without being affected by gene silencing or negative insertion site effect. Generally, in the future, these results could provide a new perspective on the use of CRISPR/Cas-based RNP-mediated knock-in for the development of microalgal strains producing synthetic novel biomolecules and complex recombinant proteins and accelerate the commercialization of the food-grade microorganism *C. reinhardtii*.

## Data availability statement

The datasets presented in this study can be found in online repositories. The names of the repository/repositories and accession number(s) can be found below: https://www.ncbi.nlm.nih.gov/genbank/, OP566529. https://www.ncbi.nlm.nih.gov/genbank/, OP566530 https://www.ncbi.nlm.nih.gov/genbank/, OP566531 https://www.ncbi.nlm.nih.gov/genbank/, OP566532 https://www.ncbi.nlm.nih.gov/genbank/, OP595611 https://www.ncbi.nlm.nih.gov/genbank/, OP595612 https://www.ncbi.nlm.nih.gov/genbank/, OP236417 https://www.ncbi.nlm.nih.gov/genbank/, OP236418 https://www.ncbi.nlm.nih.gov/genbank/, CM008970.

## Author contributions

HZS performed the research, designed the experiments, and prepared the manuscript draft. AA designed the project and revised the manuscript. HO designed the project, supervised the experiments, analyzed the data and revised the manuscript. SK helped in the project process, analyzed the data, and edited the manuscript. All authors contributed to the article and approved the submitted version.
