# Supplementary material for: Site-specific gene knock-in and bacterial phytase gene expression in Chlamydomonas reinhardtii via Cas9 RNP-mediated HDR
Source: Front Plant Sci. 2023 May 19;14:1150436. doi: 10.3389/fpls.2023.1150436 (PMC10235511; doi:10.3389/fpls.2023.1150436)
Supplement: Supplementary file 1 [file DataSheet_1.pdf]

1 Supplementary Figures

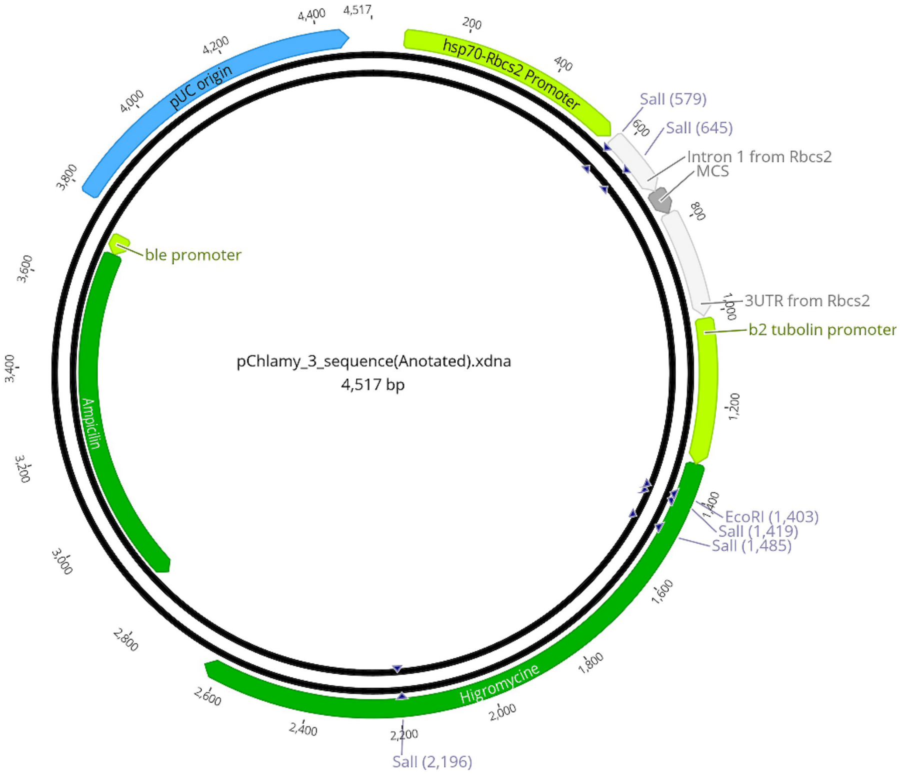

Figure 1. pChlamy 3 backbone

Supplementary Figure 1

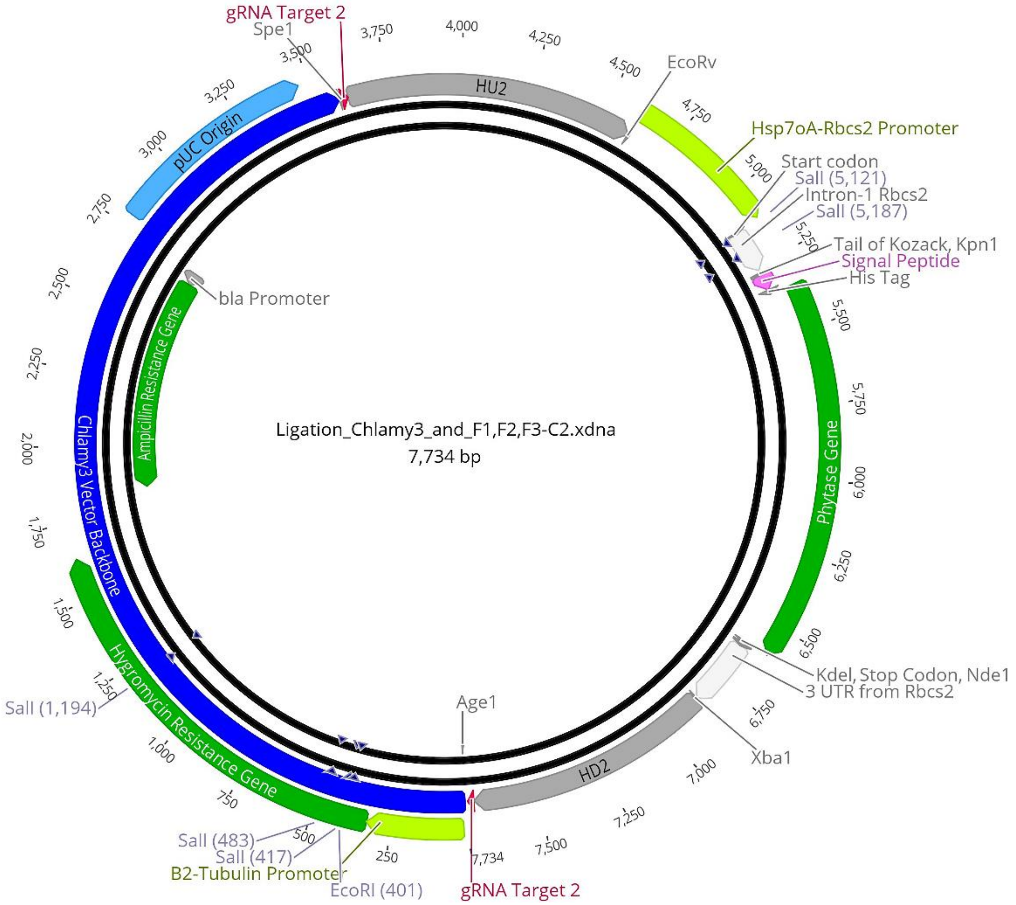

Figure 2. Donor plasmid "p1ChlamyCK-I/O" used in this study for HDR knock-in of the *Nit1* gene in *C. reinhardtii* (Genbank Accession number: OP236418) containing: The segments of **pChlamy3 vector backbone** including: B2-Tubulin promoter, Hygromycin resistance gene, Ampicillin resistance gene and bla promoter. **(F1) Left homologous arm** "HU2" containing: Spe1 restriction site, gRNA target2, PAM, Left homology arm Sequence and EcoRv restriction enzyme. **(F2) Inner construct** containing: HSP70A-Rbcs2 promoter, Rbcs2 Intron1, Signal peptide, His tag, Phytase gene, KDEL and Rbcs2 3UTR. **(F3) Right homologous arm** "HD2" containing: Xba1 restriction site, Right homology arm sequence, gRNA target2, PAM and Age1 restriction enzyme.

Supplementary Figure 2

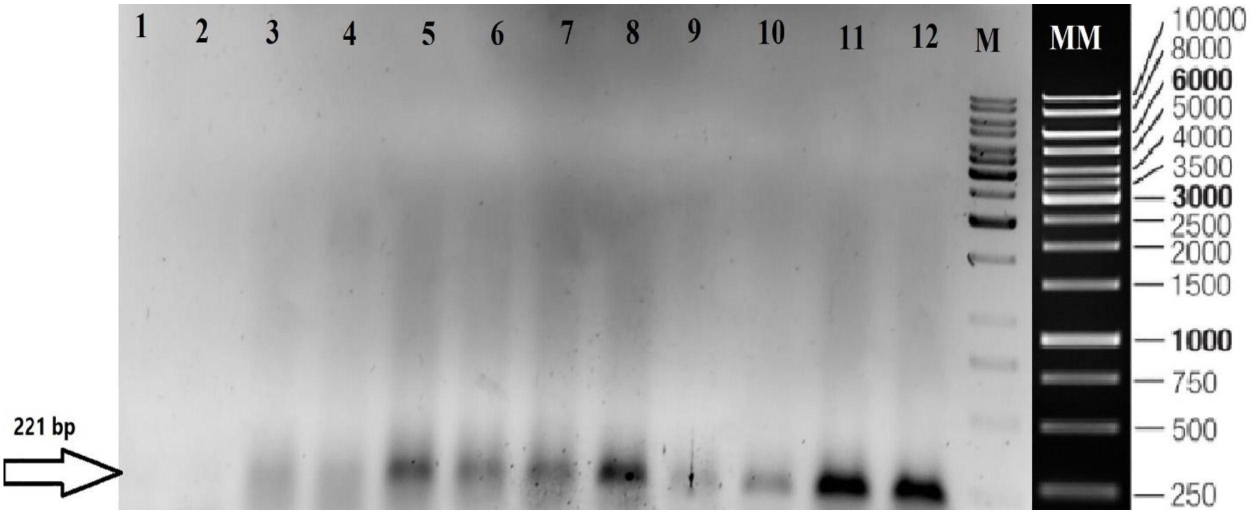

Figure 3. TBE Agarose gel (1%) showing qRT-PCR products including: **(1)** NTC [Non-Template Control] using Fq-Phytase, Rq-Phytase primer to confirm phytase gene expression; **(2)** NTC with using F-CBLP, R-CBLP primer to confirm reference gene expression; **(3)** Sample colony number 332 [considered as control sample (transformation without using RNP complex and only using donor plasmid "p1ChlamyCK-I/O")] using Fq-Phytase, Rq-Phytase primer to confirm phytase gene expression; **(4)** Sample colony number 332 using F-CBLP, R-CBLP primer to confirm reference gene expression; **(5)** Sample colony number 152 [Correct editing sample (transformed by RNP complex and donor plasmid "p1ChlamyCK-I/O")] using Fq-Phytase, Rq-Phytase primer to confirm phytase gene expression; **(6)** Sample colony number 152 using F-CBLP, R-CBLP primer to confirm reference gene expression; **(7)** Sample colony number 81 [Correct editing sample (transformed using RNP complex and donor plasmid "p1ChlamyCK-I/O")] using Fq-Phytase, Rq-Phytase primer to confirm phytase gene expression; **(8)** Sample colony number 81 using F-CBLP, R-CBLP primer to confirm reference gene expression; **(9)** Sample colony number 153 [Correct editing sample (transformed by RNP complex and donor plasmid "p1ChlamyCK-I/O")] using Fq-Phytase, Rq-Phytase primer to confirm phytase gene expression; **(10)** Sample colony number 153 using F-CBLP, R-CBLP primer to confirm reference gene expression; **(11)** Sample colony number 215 [Correct editing sample (transformed by RNP complex and donor plasmid "p1ChlamyCK-I/O")] using Fq-Phytase, Rq-Phytase primer to confirm phytase gene expression; **(12)** Sample colony number 215 using F-CBLP, R-CBLP primer to confirm reference gene expression; **(M)** DNA size marker (GeneRuler 1 kb DNA Ladder, ready-to-use (Catalog Number SM0313)); **(MM)** Marker Map of GeneRuler 1 kb DNA Ladder, ready-to-use (Catalog Number SM0313).

Supplementary Figure 3

2 Supplementary Tables

Supplementary Table 1

Table 1. Target sequences used for gRNA design. The targets that are associated with potential off-target sites bearing 1-2 mismatches compared to the on-target were avoided. Targets with GC content ranging from 30 to 70% and higher out-of-frame scores are recommended.

Rank	Target sequence	Genomic location	strand	GC content (%)	Self-complementary	MM 0	MM 1	MM 2	MM 3	Efficiency
1	AAGAGCATTGGC TTCAACTGGGG	SEQ:245	+	45	0	1	0	0	0	63.90
2	TCGTCCATGGTCA AAGTCAACGG	SEQ:140	-	45	1	1	0	0	0	60.51
3	TTGCGCACGAAG TGTACGGCCGG	SEQ:50	-	60	0	1	0	0	0	57.91
4	GCGTGATGAAAC CGTACTGTTGG	SEQ:25	-	50	1	1	0	0	0	56.95
5	CCACCTACTGGA CGGGCGTGCGG	SEQ:285	+	70	2	1	0	0	0	57.86
6	CGCGACCTGTTG CAGCACGCCGG	SEQ:311	+	70	0	1	0	0	0	55.69
7	ACTGTTGGTTTGT GTAGGGTTGG	SEQ:10	-	45	0	1	0	0	0	53.51
**8**	**CCCTACACAAAC CAACAGTACGG**	**SEQ:14**	**+**	**45**	**0**	**1**	**0**	**0**	**0**	**53.46**
9	CAAGCCGTTGAC TTTGACCATGG	SEQ:136	+	50	2	1	0	0	0	52.97
10	GACTTTGACCAT GGACGAGCTGG	SEQ:145	+	55	2	1	0	0	0	52.34
11	AGAAGAGCATTG GCTTCAACTGG	SEQ:243	+	45	1	1	0	0	0	41.16
12	GTCACCGGGAAG GTGACGGAGGG	SEQ:176	-	65	0	1	0	0	1	65.50
13	GCGACGAAGTGT ACGGCCGGCGG	SEQ:47	-	70	0	1	0	0	1	61.02
14	GTACACTTCGTGC GCAACCACGG	SEQ:56	+	55	0	1	0	0	1	60.10

Supplementary Table 2

Table 2. List of primers used in the present study for PCR and MOE PCR to amplification and assembling different segments.

Primer name	Sequence (5'-3')
F-gr2	AGCTAATACGACTCACTATAGGCCCTACACAAACCAACAGTAGTTTTAGAGCTAGAAATAGC
R-gr123	AAAAGCACCGACTCGGTGCCACTTTTTCAAGTTGATAACGGACTAGCCTTATTTTAACTTGCTATTTCTAGCTCTAAA
F-SE1	ATTATAGGTACCGTCCACCCGCGCCCCGCTGCTGGCCCTGCTGGCCCTGCTGTG
R1-SE1	GTGTCGTTGTGGTGGTGGTGGTGGTGCGCGCGGGCGGAGCCGGCGCACAGCAGGGCCAGCAGG
R2-SE1	GGATGACCACCTTCTCCACCTGGTAGCCGGAGGCGGGCGTGTCGTTGTGGTGGTGGTG
R-SE2	GGGAGCTCGGGACTTCCATATGTCACAGCTCGTCCTTCTGCAGCTGGCAGCCCGGCTCCACGGACTGGCTC
F-INC2	CCTCCAATCCCCCAAACCAGATATCTCGCTGAGGCTTGACATGATTG
R-INC2	GCCGTGGTTGCGCACGAAGTGTACTCTAGACGCTTCAAATACGCCCAGC
F-HU2	GACCGAGCGCAGCGAGTCAGTGAGCGAGGAAGACTAGTCCCTACACAAACCAACAGTACGGCGCTGCATTGTGGC TTGAAG
R-HU2	CAATCATGTCAAGCCTCAGCGAGATATCTGGTTTGGGGGATTGGAGG
F-HD2	GCTGGGCGTATTTGAAGCGTCTAGAGTACACTTCGTGCGCAACCACG
R-HD2	GAAAGACATTCACCGGTCCGTACTGTTGGTTTGTGTAGGGCCTGTGGTTCTGCTTGTATGCG
F-pCh2	GCCCTACACAAACCAACAGTACGGACCGGTGAATGTCTTTCTTGCGCTATGACACTTCCAGCAAAAGGTAGG
R-pCh2	GGGACTAGTCTTCCTCGCTCACTGACTCGCTGCGCTCGGTCG
T12-F	CGAATCCTGCCAGTATGACC
T12-R	AGCCAATGCTCTTCTTCAGC
Fq-Phytase	AACCTGAACCAGCGCTACAT
Rq-Phytase	GCGTACTCCAGCAGGAAGAT
F-CBLP	GGCTGGGACAAGATGGTCAAG
R-CBLP	GAGAAGCACAGGCAGTGGAT

Supplementary Table 3

Table 3. PCR reaction ingredient and program for gRNA DNA template (gDNA) synthesis.

Component	volume		Temperature	Time	
Primer **F-gr2** (100 µM)	1 µl		95 °C	4 min	
Primer **R-gr123** (100 µM)	1 µl		95 °C	20 sec	
5x HF buffer	10 µl	PCR program	50 °C	Temperature Ramp: 0.1°C /sec from 50°C to 48°C	25 cycle
10 mM dNTP	2.5 µl		48 °C	10 sec	
Phusion DNA polymerase	0.7 µl		68 °C	90 sec	
Distilled water	35 µl		68 °C	7 min	
total	50 µl		4 °C	15 min	

Supplementary Table 4

Table 4. PCR reaction ingredients and program for assembling of phytase gene (Part 1).

Reagent	Volume		Temperature	Time	
Primer **F-SE1** (50 µM)	1 µl				
Primer **R1-SE1** (50 µM)	1 µl		98 °C	4 min	
Primer **R2-SE1** (50 µM)	1 µl	PCR program	98 °C	30 sec	
5x HF buffer	10 µl		57 °C	20 sec	25 cycles
2 mM dNTP	5 µl		68 °C	1 min	
Phusion DNA polymerase	1 µl		68 °C	7 min	
Distilled water	31 µl		4 °C	15 min	
total	50 µl				

Supplementary Table 5

Table 5. PCR reaction ingredients and program for assembling of phytase gene (part 2).

Component	Volume		Temperature	Time	
PCR product from phytase gene (part 1)	2 µl (30ng)				
Primer **R-SE2** (100 µM)	1 µl	PCR program	98 °C	4 min	
Synthesized phytase gene	1 µl (30 ng)		98 °C	30 sec	35 cycles
5x HF buffer	10 µl		68 °C	90 sec	
2 mM dNTP	5 µl		68 °C	5 min	
Phusion DNA polymerase	1 µl		4 °C	15 min	
Distilled water	35 µl				
total	50 µl				

Supplementary Table 6

Table 6. Multiple overlap extension PCR (MOE-PCR) assembly of the donor plasmid "p1ChlamyCK-I/O" (Accession number: OP236418).

Component	Volume		Temperature	Time	
(PCR product) Vector backbone	2.3 µl (200 ng)				
(PCR product) Left Homologous arm PCR	4.2 µl (112 ng)				
(PCR product) Right Homologous arm PCR	6.2 µl (112 ng)				
(PCR product) Inner construct	10 µl (257 ng)	PCR program	95 °C<br>98 °C<br>68.4 °C<br>68 °C<br>4 °C	5 min<br>20 sec<br>250 sec<br>10 min<br>15 min	40 cycles
5x HF buffer	10 µl				
2 mM dNTP	10 µl				
Phusion DNA polymerase	1.2 µl				
Distilled water	6.1 µl				
total	50 µl				

Supplementary Table 7

Table 7. Primers used for sequencing by primer walking of the donor plasmid "p1ChlamyCK-I/O" (Accession number: OP236418).

Primer name	Sequence (5'-3')
41F	CGCTATGACACTTCCAGCAA
309F	GCCTCTTCCTCTTCGTTTCA
829F	GGCCCTACCTGGTGATGAG
1328F	AAGCGGACCGAGGACTTC
1875F	GGCACCTATCTCAGCGATCT
2311F	ACTCATGGTTATGGCAGCA
2804F	CGTCAGACCCCGTAGAAAAG
3327F	GGGAAACGCCTGGTATCTTT
3833F	TGAACTTGCGTCTCTCTTCG
4305F	CGTGCTCATGCAGGTGAG
4806F	ACGGTGACCTCCACTTTCAG
5271F	CCATTTGCAGGAGATTCGAG
5810F	GTGGAGAAGGAAGCCCAGAC
6390F	GGCAAGCAGTACGTGAGC
6813F	ACGATCCTCCGTTGATTTTG
7320F	GCGTATAACCGTGGGAAGAG
121R	GTCGAAGCATCATCGGTGT

Supplementary Table 8

Table 8. Optimization of the in vitro Cas9 cleavage assay.

Components	Experiment 1	Experiment 2	Experiment 3	Experiment 4	Experiment 5	Experiment 6	Experiment 7
gRNA	0.5 µl (162.5 ng)	1 µl (325 ng)	2 µl (650 ng)	4 µl (1300 ng)	2 µl (650 ng)	2 µl (650 ng)	2 µl (650 ng)
Cas9	0.5 µl (16.25 ng)	1 µl (32.5 ng)	2 µl (65 ng)	4 µl (130 ng)	2 µl (65 ng)	-	-
Activity buffer (HEPES 200mM, NaCl 1M, MgCl2 50mM, EDTA 1mM with pH: 6.5)	3 µl	3 µl	3 µl	3 µl	3 µl	3 µl	3 µl
Nuclease free water	10.5 µl	9.5 µl	7.5 µl	3.5 µl	4 µl	8 µl	9.5 µl
Incubation at 37℃- 15 min							
Donor Plasmid	400 ng	400 ng	400 ng	400 ng	-	-	400 ng
PCR product of NR gene (Exon 1 and 2)	-	-	-	-	400 ng	400 ng	-
Incubation at 37℃- 4 h							
Incubation at 65℃- 10 min							

Supplementary Table 9

Table 9. Transformation and editing efficiency: **(A)** Selected hygromycin resistance colonies; **(B)** Phytase Positive Colonies; **(C)** Colonies containing plasmid residues [Incorrect Edition]; **(D)** Colonies containing Correct Editing [Knocked-in phytase gene at the desired position (NR gene–Exon2)].

Experiment (Repeat)	A	B	C	D
1 (1)	32	20	6	6
1 (2)	13	9	-	4
2 (1)	12	6	1	1
2 (2)	18	10	3	3
3 (1)	53	40	10	7
3 (2)	34	21	8	3
Total	162	106	28	24

Supplementary Table 10

Table 10. qRT-PCR analysis (CT values): **(A)** NTC 1 [Non-Template Control for CBLP reference gene]; **(B)** NTC 2 1 [Non-Template Control for Phytase gene]; **(C)** CBLP reference gene in UVM11 *C. reinhardtii* strain; **(D)** Phytase gene in UVM11 C. reinhardtii strain; **(E)** CBLP reference gene in sample 215 [correct edited sample]; **(F)** Phytase gene in Sample 215 [correct edited sample]; **(G)** CBLP reference gene in sample 153 [correct edited sample]; **(H)** Phytase gene in Sample 153 [correct edited sample]; **(I)** CBLP reference gene in sample 81 [correct edited sample]; **(J)** Phytase gene in Sample 81 [correct edited sample]; **(K)** CBLP reference gene in sample 332 [considered as control sample (transformation without using RNP complex and only using donor plasmid “p1ChlamyCK-I/O”)]; **(L)** Phytase gene in Sample 332 [considered as control sample (transformed without RNP complex and only using donor plasmid “p1ChlamyCK-I/O”)].

Sample	A	B	C	D	E	F	G	H	I	J	K	L
CT Average	38	38	23	37	23	23	23	24	24	24	24	29
